# *Helicobacter pylori* Pathogenicity Factors Related to Gastric Cancer

**DOI:** 10.1155/2017/7942489

**Published:** 2017-12-17

**Authors:** Kianoosh Dadashzadeh, Maikel P. Peppelenbosch, Akyala Ishaku Adamu

**Affiliations:** ^1^Department of Medical Laboratory Sciences, Marand Branch, Islamic Azad University, Marand, Iran; ^2^Erasmus MC Cancer Institute, Erasmus University Rotterdam, Rotterdam, Netherlands

## Abstract

**Background:**

Although the causal relationship between* Helicobacter pylori* infection and the development of gastric cancer is firmly established, the exact nature of the pathogenicity factors of* H. pylori* that predispose to gastric oncogenesis remains incompletely characterized. We investigated the association between* H. pylori *virulence genotypes and disease in a well-characterized cohort consisting of 109* H. pylori* isolates from gastric biopsies originating from patients.

**Methods:**

The prevalence of genotype was assessed by PCR and related to clinical histopathological parameters.

**Results:**

The relation of* bab ***A2** and* bab ***B** negative and* ice ***A1** positive genotype as a single genotype and the development of cases to GC was statistically significant (*P* < 0.001). The* cag ***E**,* cag ***A**, and* ice ***A1 **were found more commonly in patients with GC as compared with the other groups. The relation of the presence of* ice ***A1** and the development of cases to GC was statistically significant (*P* = 0.008), but* bab ***A2** and* bab ***B **alleles were not detected in these patients. These apparent negative associations were still statically significant (*P* = 0  and 0.005).

**Conclusion:**

Our results show an elevated prevalence of infection with* H. pylori* strains carrying known virulence genotypes with high genetic diversity. This highlights the importance of identifying gene variants for an early detection of virulent genotypes.

## 1. Introduction

Gastric cancer remains a prevalent disease worldwide with a poor prognosis.* Helicobacter pylori* plays a major role in gastric carcinogenesis*. H. pylori* colonization leads to chronic gastritis, which predisposes to atrophic gastritis, intestinal metaplasia, dysplasia, and eventually gastric cancer. Screening, treatment, and prevention of* H. pylori* colonization can reduce the incidence of gastric cancer [1]. Other interventions that may yield a similar effect, although of smaller magnitude, include promotion of a healthy lifestyle including dietary measures, nonsmoking, low alcohol intake, and sufficient physical activity [2]. Furthermore, increasing evidence suggests that host factors, including genetic make-up, are also important determinants for carcinogenesis in* H. pylori* infection [3]. Colonization of* H. pylori* has been associated with chronic gastritis because it can trigger fulminant inflammatory response which can lead to pathological conditions such as gastric carcinoma and peptic ulcer disease [4]. Consequently a clinical need exists to stratify* H. pylori*-infected patients with respect to their propensity to develop* H. pylori*-related pathology; especially for GC this is felt as a pressing concern. The importance of* H. pylori* virulence factors is evident from the serious clinical outcome associated with bacteria positive for the vacillating cytotoxic (*vac ***A**) and the cytotoxin-associated gene A (*cag ***A**) antigen [5]. However, these two virulence factors are insufficient to explain the variety in clinical presentation of pathology associates with* H. pylori* infection [6, 7]. A potential* H. pylori* virulence factor possibly important to explain this variance in clinical outcome is the cag pathogenicity island (cag-PAI). The* cag ***A** gene is a marker for the presence of the cag-PAI of approximately 40 kb, whose presence is associated with the more severe clinical outcomes [8, 9]. A type IV secretion system translocates* cag ***A** protein into gastric epithelial cells, where it is phosphorylated. When this modification occurs,* cag ***A** affects various cellular processes and signal transduction pathways, such as disruption of tight and adherent junctions that lead to proinflammatory and mitogenic responses: effects [8, 10]. One of the six cag-PAI genes is* cag ***E**, located in the right half of the cag-PAI, that has been shown to induce secretion of interleukin- (IL-) 8, from infected host epithelial cells [11, 12].

Another putative virulence factor is* ice ***A**, whose gene has two main allelic variants,* ice ***A1** and* ice ***A2**. The expression of iceA1 is upregulated on contact of* H. pylori* with human epithelial cells and may be linked with peptic ulcer disease [16, 17]. The blood group antigen binding adhesin (*bab ***A**), a 78 KDa outer membrane protein encoded by the babA2 gene, binds to Lewis b antigens and ABO antigen [18, 19]. Although three* bab* alleles have been identified (*bab ***A1**,* bab ***A2**, and* bab ***B**), only the* bab ***A2** gene product is functionally active [20]. Studies in Western populations have associated the presence of the* bab ***A2** gene with gastric cancer [19, 21]. The aim of this study is to assess the genotype of* H. pylori* strains infecting patients with chronic gastritis through the evaluation of the prevalence of several genes coding for virulence factors.

## 2. Materials and Methods

This research was approved by the regional Medical Research Ethics Committee of Azad University of Medical Science on 19 Jul 2016 (number 1311/28772) and all patients provided written informed consent for this research.

This study protocol conforms to the ethical guidelines of the 1975 Declaration of Helsinki as reflected in a prior approval by the institution's human research committee.

### 2.1. Patients, Bacterial Strains, and Cultivation

Our cohort consisted of 109 clinical isolates of* H. pylori; *49 (45%) were from females and 60 from males (55%).

From male patients (sex ratio F/M: 0.82), the average age of the patients from which the isolates were obtained was 39 ± 17 years.* H. pylori* was isolated employing gastric biopsies of patients presenting with gastritis, peptic ulcer, or gastric cancer. Patients were not exposed to antimicrobial agents at least one week before endoscopy was performed. Following collection, gastric biopsy samples were homogenized and cultured onto Brucella agar supplanted with 5% sheep blood and antibiotics (vancomycin, amphotericin B, and trimethoprim). Culture plates were incubated at microaerophilic condition, 37°C and high humidity for 5–7 days. Organisms were identified as* H. pylori* based on colony morphology, gram staining, and positive oxidase, catalase, and urease tests.

### 2.2. DNA Extraction from Culture

Genomic DNA of total* H. pylori* isolates was extracted using the QiAamp DNA mini kit (QIAGEN, Hilden, Germany) according to manufacturer's protocol and stored at −20°C until use.

### 2.3. Detection of Genes

In this study, PCR was used to detect the* H. pylori* specific* ureC* gene for confirming that the cultures represented a* bona-fide H. pylori *isolate, and the same technique was employed to establish presence or absence of* cag ***E**,* cag ***A**,* ice ***A1**,* ice ***A2 ***bab ***A2**, and* bab ***B** genes.

All primer sets were selected from published literature (listed in Table 1). PCR reactions were performed in a volume of 50 *μ*L containing 10 mmol/L Tris-HCl, 1.5 mmol/L MgCl_2_, 0.2 mmol/L of each deoxynucleotide, 25 pmol of each primer, and 2.5 units of Taq polymerase (Geneone, Germany) [22, 23]. The thermal cycler program used consisted of the following steps: initial denaturation at 94°C for 3 min followed by 35 cycles of 30 seconds at 94°C (denaturation); 30 seconds at 58°C for* cag ***A** and* glm ***M** [24]; 30 seconds at 53°C for* cag ***E**; 48°C for* bab ***A2**; 49°C for* bab ***B**; 57°C for* ice ***A1**; and 48°C and for* ice ***A2** all annealing steps, followed by 30 seconds at 72°C (extension step); and a final extension step was 3 min at 72°C [25].

### 2.4. Statistics Analysis

Data were analyzed by SPSS version 16. Fisher's exact test or the Chi-square test was used for analysis of categorical data. A *P* value of <0.05 was considered statistically significant.

## 3. Results

### 3.1. Description of the Patient Cohort and Isolation of Study Samples

A total of 109* H. pylori* samples were isolated from an initial number of 359 biopsies included in this study (yielding an apparent infection rate of 30.4%), of which 60 (55%) were derived from male patients and 49 (45%) isolates came from female patients (sex ratio F/M: 0.82). The average patients' age at the time of endoscopy was 39 ± 17 years. From the 109 patients with established* H. pylori* infection, 81 patients had presented with a nonulcer dyspepsia, nineteen patients with a peptic ulcer dyspepsia, and nine patients with GC (Signet Ring Cell Carcinoma (SRCC)). There was no significant difference between the mean age and sex of patients with and without ulcers and cancer. We concluded that our cohort would enable analysis of the link between* H. pylori *virulence factors and clinical presentation.

### 3.2. The Presence of Virulence Factors Is Common in the Clinical Cohort

The PCR-based amplification showed that the* cag ***E**,* bab ***A2**, and* bab ***B** positive strains had a prevalence of, respectively, 55.9%, 71.7%, and 61.3% in our cohort, whereas* cag ***A**,* ice ***A1**, and* ice ***A2** were detected in, respectively, 70.6%, 42.2%, and 13.2% of the patients included in this study (Table 2). Thus, the virulence factors selected for this study are commonly detected in our patient cohort and allow statistical analysis as to their relation to disease manifestation.

### 3.3. Specific* H. pylori* Virulence Factors Show Trends of Being Positively or Negatively Associated with Patient's Clinical Presentation

In our study the frequency of* cag ***E**-positive isolates obtained from patients with nonulcer dyspepsia, peptic ulcer dyspepsia, or GC patients was 49.4%, 68.4%, and 88.9%, respectively, but this apparent positive association with more severe disease did not reach statistical significance (*P* = 0.198). The* bab ***A2** genotype was detected in 74%, 84.2%, and 0% of isolates from NUD, PUD, and GC patients. The percentage of* bab ***A2**^−^ genotype within GC patients was significantly higher than that of* cag ***A**^+^ genotype (*P* < 0.001).

Also the percentage of* bab ***B**^**+**^ was 65.4% and 63.1% for NUD and PUD cases, respectively, but for isolates from patients with GC (0%), this apparent negative association was statistically significant (*P* = 0.005). Conversely the prevalence of the* ice ***A2** allele was observed in NUD patients (13.6%) and in PUD (15.8%) and in GC patients (0%), but no significant association was observed between* ice*A2 genotype and GC (*P* = 0.779). Similarly, in this study the distribution of* ice ***A1** and clinical outcome was analyzed statistically and it was observed that the frequency of* ice ***A1**-positive isolates in NUD, PUD, and GC patients was 35.8%, 42.1%, and 100%, respectively. The relation of the presence of* ice ***A1** and the development of cases to gastric cancer was statistically significant (*P* = 0.008).

Although the* cag ***A** allele was observed in NUD (65.4%) cases and in PUD patients (78.9%) and in GC patients (100%), there was not statistically significant association between* cag ***A** and the gastric outcomes (*P* = 0.134). A full overview of all virulence factors studied and their linkage to specific clinical manifestations is provided through Table 2.

It is apparent from our data that* cag ***E**,* cag ***A**, and* ice ***A1** are more common in patients with gastric cancer than in the other patient groups, whereas* bab ***A2** and* bab ***B** alleles were absent in patients with GC.

### 3.4. Combining Different Virulence Factors Allows Stratification of the Patient Cohort with respect to Patient Stratification

We examined eight different combinations based on analysis of* bab ***A2**,* bab ***B**, and* ice ***A1** genotypes (positive and negative) in patients as a single genotype (Table 3). We were able to identify an association between these genotypes and clinical outcome. The frequency distributions of the combination genotypes of* H. pylori* showed the relation of* bab ***A2** and* bab ***B** negative and* ice ***A1** positive genotype and the development of cases to gastric cancer was statistically significant (*P* < 0.001) among the patient groups. But there was not statistically significant association between other genotype combinations and the gastric cancer (*P* ≥ 0.113).

The apparent absence of* bab ***A2** and* bab ***B **alleles in GC patients raises, however, hopes that larger studies may establish the usefulness of these alleles in guiding patient management.

## 4. Discussion

Various studies have observed substantial differences in incidence and/or severity of gastroduodenal pathologies related to* H. pylori* which may vary according to geographical regions [26]. Although many factors may contribute to these differences, an obvious contributing factor is the different distribution of pathogenic markers in circulating strains [27]. The clinical relevance of the putative virulence-associated genes of* H. pylori* and geographical region remains controversial. Other factors that influence the risks for atrophy and cancer in the presence of infection may be related to the time when infection occurred, to other environmental factors, and to the host genetic variation [28]. In particular single nucleotide polymorphisms in genes that influence bacterial handling via pattern recognition receptors appear to be involved, further strengthening the link between host risk factors,* H. pylori* incidence, and cancer [29]. In the present study we exploited this situation to study the relationship between selected virulence genes of* H. pylori* and the clinical status. Our results clearly support the notion that further studies aimed at establishing the negative predictive value of the presence of* bab ***A2** and* bab ***B **alleles for GC development are warranted.

The* cag ***E** is a pathogenicity biomarker of* H. pylori*. A survey of previous studies suggested that the* cag ***E** prevalence is different around the world [30]. The general importance of* cag ***E** is best illustrated by its high frequency in GC patients as demonstrated by studies performed in patient populations derived from India (100%), Turkey (81.8%), and Thailand (93.8%) [31]. However, the prevalence of* cag ***E** gene in this study was only 55.9%, markedly different from the results obtained in the aforementioned countries [32]. In the present study* cag ***E**-positive isolates were slightly more detected in isolates from peptic ulcer dyspepsia (PUD) patients (68.4%), but the potential significance of this finding, if any, remains to be established. The prevalence of* cag ***A** gene in our cohort is 70.6%, which resembles the situation in Western countries (Yamaoka et al., 2002), but is markedly lower than that observed in East Asian countries where* cag ***A** is present in more than 90% of cases [32]. Like* cag ***E**,* cag ***A**-positive isolates were enriched in samples obtained from peptic ulcer dyspepsia patients, albeit not in a statistically significant manner. Nevertheless, our findings support previous studies [32] (Wu et al., 2003) and should prove interesting in including our data in a meta-analysis of virulence genes in this respect. Our results show that the prevalence of* ice ***A1** and* ice ***A2** genes in isolates was 42.2% and 13.2%, respectively. These results are in agreement with previous studies showing that the* ice ***A1** gene is prevalent in Japanese, Korean, and Dutch patients [13] (Ito et al., 2000; Shiota et al., 2013); conversely the* ice ***A2** allele was predominant in the United States and Colombia. In our cohort, the relation of the presence of* ice ***A1** and the development of cases to gastric cancer was statistically significant (*P* = 0.008). But our data showed that there was no significant association between* ice ***A2 **and GC compared with PUD or NUD. However, several studies have reported different results, as the* ice ***A2** gene was detected to be predominant genotype in these studies (Aghdam et al., 2014; Biernat et al., 2014).

The* bab ***A** is one of the mediators for the attachment to gastric cells by* H. pylori* [33]. More recent analysis of* bab ***A2** as a virulence marker has produced conflicting data on the usefulness of* bab ***A2** expression in predicting clinical outcome, which is most likely dependent on the geographic origin of the* H. pylori* strains. Survey of previous studies on Portuguese, Thai, and India populations showed that* bab ***A2** is not a marker for peptic ulcer disease or gastric cancer [12, 26, 34]. However, several studies have reported different results for strains isolated from Turkey or Germany [26, 35]. Our study suggests that* bab ***A2**, “although quite prevalent,” is associated with reduced propensity to develop GC and is thus associated with less oncogenic* H. pylori. *Similar results were obtained concerning* bab ***B**.

Our results showed the relation of* bab ***A2** and* bab ***B** negative and* ice ***A1** positive genotype and the development of cases to gastric cancer was statistically significant. Further studies are required to determine the functions of* bab ***A2** and* bab ***B** and their relationship with disease outcome and whether the presence of these alleles indicates the presence of bacteria unlikely to confer progression towards GC.

## Figures and Tables

**Table 1:** Primer sequences for polymerase chain reaction.

Gene	Primer	Nucleotide sequence	Size (bp)	Reference
*ure * **C** (*glm ***M**)	Hp-F	GGATAAGCTTTTAGGGGTGTTAGGGG	294	[13]
	HP-R	GCTTACTTTCTAACACTAACGCGC		
*cag * **E**	*cag * **E**-F	TTGAAAACTTCAAGGATAGGATAGAGC	508	[12]
	*cag * **E**-R	GCCTAGCGTAATATCACCATTACCC		
*bab * **A2**	*bab * **A2-**F	CCAAACGAAACAAAAAGCGT	271	[6]
	*bab * **A2-**R	GCTTGTGTAAAAGCCGTCGT		
*bab * **B**	*bab * **B** -F	ATGAAAAAAACCCTTTTAC	496	[14]
	*bab * **B**-R	CGAATTGCAAGTGATGGT		
*cag * **A**	*cag * **A**-F	AGGGATAACAGGCAAGCTTTTGA	352	[15]
	*cag * **A**-R	CTGCAAAAGATTGTTTGGCAGA		
*ice * **A1**	*ice * **A1**-F	GTGTTTTTAACCAAAGTATC	247	[13]
	*ice * **A1**-R	CTATAGCCASTYTCTTTGCA		
*ice * **A2**	*ice * **A2**-F	GTTGGGTATATCACAATTTAT	229	[13]
	*ice * **A2**-R	TTRCCCTATTTTCTAGTAGGT		

**Table 2:** Relationship between clinical outcome and status of *cag ***E**, *bab ***A2**, *bab ***B***, ice ***A1**, *ice ***A2**, and *Cag ***A**.

Genotypes	Number (%) of isolates			Total (*n* = 109)	*P* value
	NUD (*n* = 81)	PUD (*n* = 19)	GC (*n* = 9)		
*cag * **E**	40 (49.4%)	13 (68.4%)	8 (88.9%)	61 (55.9%)	0.198
*bab * **A2**	60 (74%)	16 (84.2%)	0	76 (71.7%)	0.001
*bab * **B**	53 (65.4%)	12 (63.1%)	0	65 (61.3%)	0.005
*ice * **A1**	29 (35.8%)	8 (42.1%)	9 (100%)	46 (42.2%)	0.008
*ice * **A2**	11 (13.6%)	3 (15.8%)	0	14 (13.2%)	0.779
*cag * **A**	53 (65.4%)	15 (78.9%)	9 (100%)	77 (70.6%)	0.134

NUD: nonulcer dyspepsia; PUD: peptic ulcer disease; GC: gastric cancer; *P*v: *P* value.

**Table 3:** Combination of *bab ***A2**, *bab ***B**, and *ice ***A1 **genotypes and clinical outcome.

*bab * **A2**	*bab * **B**	*ice * **A1**	**GC ** (*n* = 9)	**NUD **(*n* = 81)	**PUD **(*n* = 19)	**Total **(*n* = 109)	*P* value
Positive	Positive	Positive	0	19	6	25	0.113
Positive	Positive	Negative	0	15	3	18	0.121
Positive	Negative	Positive	0	12	2	14	0.136
Positive	Negative	Negative	0	9	4	13	0.196
Negative	Positive	Positive	3	7	1	11	0.216
Negative	Positive	Negative	0	11	2	13	0.157
Negative	Negative	Positive	6	3	1	10	0.001
Negative	Negative	Negative	0	5	0	5	0.253

Presence of a gene = positive; absence of a gene = negative; *P*v: *P* value.
